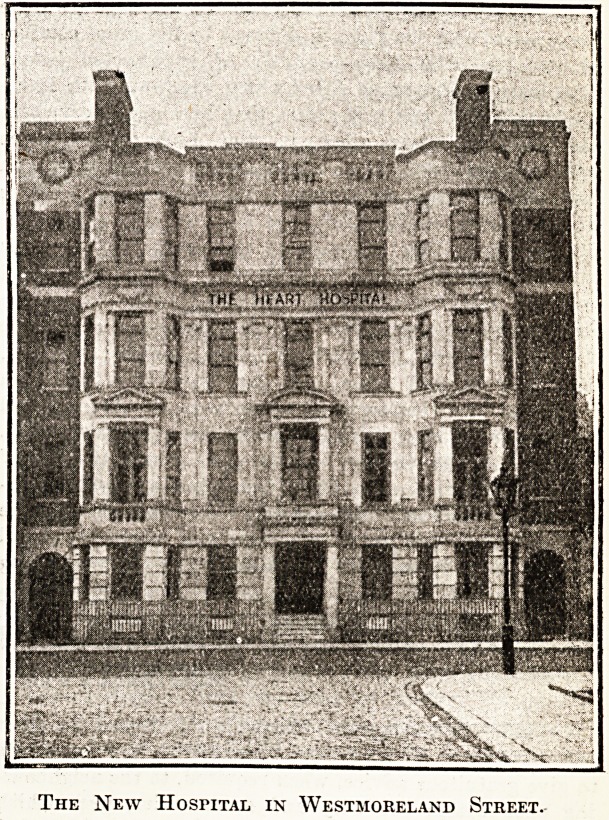# The New Institution for Diseases of the Heart

**Published:** 1914-01-17

**Authors:** 


### THE NEW INSTITUTION FOR DISEASES OF THE HEART.

### A Tour of the New Buildings with the Secretary.

We note elsewhere in this issue the opening last Monday by Prince Arthur of Connaught of the new buildings of the National Hospital for Diseases of the Heart, of which we publish below an illustration. Our readers will remember that the institution, which was first established in 1857, moved from its old quarters in Soho Square, where for upwards of forty years it has inhabited the beautiful old house associated with the name of Sir Joseph Banks, a fine example of eighteenth-century domestic architecture, containing at the same time some Adams ceilings and mantelpieces. These merits, however, are at a discount in a modern hospital, and some three years ago, as Sir James Harrison, the Chairman, expressed it to our representative, it was recognised to be "absolutely impossible" for the hospital to remain there any longer. The new buildings are in Westmoreland Street, Marylebone, and by the courtesy of Mr. Robert G. E. Whitney, the secretary, our commissioner made a tour of inspection, and heard from him the chief points of interest in the new hospital.

Passing into the board room, which is on the left of the main entrance, Mr. Whitney explained how absolutely necessary it had become for the hospital to move to new quarters. "The No. 32 Soho Square house," he began, "was built in 1785, and beautiful though it is, was as unsuitable for a hospital as anything now could be. But leaving it was not an easy matter. We had fortunately nearly come to the end of our lease, but, on the other hand, we were encumbered with heavy liabilities for dilapidation. By great good fortune and, we think we may add, a little by good management, the æsthetic virtues of the old house have proved also of commercial value, and the remainder of our lease has been disposed of to a firm of antique furniture dealers, who have taken it off our hands for their show rooms, and in addition have accepted all our liability for dilapidations. This means that we leave our old house as it were debt free, and have been able to make use of the previously created 'dilapidation fund' for the purposes of the present new building. The architect is Mr. Harold Gorlett, F.R.I.B.A., who has acted in consultation with Messrs. Percy Adams and Charles Holden. The front elevation, of Portland stone, reveals a ground floor, with three ward floors above it, and behind the flat roof of the latter is a fourth floor, over which again is a pathological unit. There is also a lower ground floor devoted to the out-patient unit and pathological, *x*-ray, and cardiographic departments." Mr. Whitney then led the way out of the board room through the vestibule to the staircase and lift.

### Practical Points: the Top Floor.

"This floor, as you see, overlooks the flat roof of the topmost ward floor, which is reached by descending a few steps. First of all, let me explain that the top floor is devoted to a part of the pathology department, consisting of a post-mortem room and a mortuary, together with a small room for the preservation of specimens. All these rooms are white tiled, and here, as everywhere else except in the wards, the floors are of granolithic. In the wards the floors are of pitch pine laid in strips, and while we are on these matters I may add that the doors are everywhere of teak; there is no terrazzo anywhere, and no linoleum, and all the fittings, door handles, and so forth are of aluminium, to save polishing. I have a strong prejudice against linoleum since I once had a culture made of the accretion which had collected at another institution where the linoleum joined the wall."

On the way down to the fourth floor, which is on a level with the flat roof above alluded to, I asked how a general idea of the building could be most simply given.

"It is really a square figure of eight," replied Mr. Whitney, "of which the narrow waist is caused by the roof-lights (which, of course, cannot be built over) for certain rooms in the out-patient and radiographic departments on the lower ground floor. The top flat roof, we hope, will be used by patients during the summer, and a shelter and lavatory accommodation have been provided for their benefit at either end. The three ward floors are below our feet as we stand here at the front of the building, but on leaving the flat we come to the back part of

The New Hospital in Westmoreland Street.

this floor, where the kitchen, pantries, and the maids' dining room are situated. There is also, of course, a service staircase at the back, and the iron fire-escape staircase, which communicates directly with each ward, is also worth notice."

### The Three Wards.

"How many beds will the new hospital accommodate?"

"There are three wards, with fourteen beds in each, giving a total of forty-two beds in all; but we do not intend to use this top ward at present, nor, in fact, to open it until funds permit, which will be when the building debt has been paid off.

"Each ward measures 48 by 26, and they are, I think, rather pretty owing to the irregular shape given to them by the two bay windows. The ward occupies the whole front of each floor, as represented by the breadth of the building looked at from the street. For the front and the back parts of the hospital are, as I said just now, the broadest parts, representing, in fact, squares of the eight which it resembles. Owing to the fact that each ward runs parallel to the street, there is not the same number of windows on each side, nor will there be a window between every bed; but you will notice the lightness and air space, and the way in which the bathroom and fire-escape staircase flank the ward on one side, and the sink-room and closet on the other."

"What are the special features?"

"First of all the way in which every bed can be connected by wires to the electro-cardiograph, so that where this is done heart changes can be recorded in the laboratory, in this case three floors away. In the old hospital, of course, this record could not be obtained without moving the patient. Now that is unnecessary, and the record of the condition of the heart is made automatically. A second point is the lighting; and by the simple contrivance of an inverted globe hung below the electric lights attached to the ceiling expensive fittings are done away with, and the light is diffused to the great comfort of patients, who often complain of unprotected lamps. The ward bathroom is fitted along one wall with linen-cupboards, and you will notice that here and everywhere else the old chain plug in the baths and basins has been returned to, as simpler and more cleanly than any of the newer substitutes. Behind the big ward is a small room either for one bed or two cots. The heating is by four radiators in the wards, and there is an open tiled fireplace at each end. Behind the small ward is the ward kitchen, connected with the service lift, and up a few stairs—by this means a mezzanine floor has been inserted—are the maids' quarters. Two sleep in each room, and they have here their own bathroom and lavatory accommodation. The second ward floor below is designed on precisely the same plan, except that the nurses' bedrooms are where the maids' bedrooms were above. Each nurse has a room to herself. On the first floor, again, the ward unit is the same, except that the ward kitchen is where the small ward was above, and at the back are the nurses' dining room, which projects further than any other part of the building at the rear, the matron's sitting and bed room, and the sitting room for two sisters."

"There now remains only the ground floor and the out-patient unit below it to be seen?"

### The Resident Medical Officer's Flat.

"Yes; on entering the hospital you have the board room on your left, the secretary's office behind it, and on your right the matron's office, and, completely self-contained, the resident medical officer's flat. He has a bedroom, sitting room, bathroom, and lavatory on the opposite side of the vestibule to my office; and a special and, I think, successful effort has been made to render that officer comfortable. The back of the ground floor is occupied mainly by the upper part of the out-patients' waiting hall, and here also are the porter's quarters, consisting of a bedroom, bathroom, and lavatory."

### The Out-patient Unit.

As we descended to the lower ground floor Mr. Whitney remarked, "Of course, the wards are still, as you saw, in the muddle of preparation for the opening and the reception of patients; and the out-patient hall is busy with patients already arrived. As you face the hospital," he went on, "the out-patients enter at the extreme right of the building, and pass down a slope, at the foot of which is a porter's box, where their case papers are given out before they pass into the out-patient waiting hall. On the near side of this as you enter are the registrar's office and two consulting rooms; on the far side lavatory accommodation and the exit, which is placed by the dispensary; and so up the slope and out on the left side of the building. This is the circular plan, of course, now generally adopted both in out-patient departments and laundries. The rest of the lower ground floor—that is to say, the front part underneath the vestibule and the wards above—is devoted to the special departments. Here are the pathological laboratory, the *x*-ray room, the electro-cardiograph laboratory (where three instruments are being installed), next to it are the record room and two dark rooms, behind which we come to the dispensary again and the drug store, which forms part of it. The lift and staircase take up the remaining space. That is a brief summary of the out-patient unit."

## Figures and Tables

**Figure f1:**